# Utilization of Cancer Information System for Breast Cancer Control in Lagos, Nigeria

**DOI:** 10.11604/pamj.2016.24.323.9632

**Published:** 2016-08-24

**Authors:** Omolola Salako, Alero Ann Robert, Kehinde Sharafadeen Okunade, Adeola Olatunji, Adeola Fakolade, Victor Isibor, Deborah Falode

**Affiliations:** 1 Cancer Information Service, Sebeccly Cancer Care & Support Center, Nigeria; 2 Department of Community Health, College of Medicine, University of Lagos, Nigeria; 3 Department of Obstetrics & Gynaecology, College of Medicine, University of Lagos, Nigeria; 4 Research & Development, Sebeccly Cancer Care & Support Center, Nigeria; 5 Department of Oncology, American Hospitals and Resorts, Lekki, Nigeria

**Keywords:** Breast Cancer, CIS, Health

## Abstract

**Introduction:**

There is a substantial increase in the incidence of breast cancer in Nigeria usually with the late stage presentations and subsequent poor rates of survival attributed mainly to a low level of cancer awareness and ignorance amongst patients. Cancer information system (CIS) is now assuming an emerging role in this respect.

**Methods:**

This was a descriptive study carried out over a one year period using a health communications program comprising of 3 breast help lines. An initial period of public awareness was carried out over a 3 months period after which members of the public were encouraged to call the help lines. Breast cancer information was provided and the socio-demographic characteristics and other relevant data of the callers were recorded by the information specialists.

**Results:**

A total of 294 people were reached during the study period. Majority of the callers (82%) sought information for themselves while the remaining 18% called on behalf of a loved one or friend. Majority [248 (84.3%)] of callers had no breast abnormality, 38 (13%) called to report breast abnormalities and required information on what to do and 8 (2.7%) were breast cancer patients who required information on how to live and cope as breast cancer survivors.

**Conclusion:**

The rapid growth of mobile phone use in the Nigeria has presented a unique opportunity and promise to improve cancer care. There is evidence to suggest that mHealth can be used to deliver increased health care services to the increasing population of cancer patients in Nigeria.

## Introduction

In 2012, breast cancer accounted for 27,304 new cases and 13,960 deaths [1]. There is a substantial increase in the incidence of breast cancer in Nigeria [2] with a persistence of late stage presentations to the hospitals as seen in various hospital based series [3]. Late stage presentations are associated with poor survival rates and have been attributed to a low level of cancer awareness and ignorance amongst patients [3]. Health care systems are gradually moving toward new models of care based on integrated care processes shared by different care givers and on an empowered role of the patient. Mobile technologies are assuming an emerging role in this scenario. This is particularly true in care processes where the patient has a particularly enhanced role, as is the case of cancer supportive care [4]. The Ebola crisis that occurred in Nigeria in 2014 was quickly curtailed partly because there was accurate information available to the public and vigorous and rapid public health response [5]. Efforts to educate the public will also empower them to proactively attend cancer screening, live a healthy life style and know the right place to go to, should the need arise, for a cancer related matter. Secondly, more patients can be educated on their disease, linked to resources and referred to approved cancer centres and this will significantly reduce the number of patients treated by untrained health care professionals as providing accurate and timely cancer information should be a high priority.

A Cancer Information Service (CIS) is a service created to meet the cancer information needs of the public. It may provide patient education, emotional support and counselling services to people affected by cancer, a CIS is primarily about giving good quality, one to one information about cancer in response to people’s questions [6]. A CIS can be provided by phone (mobile health), email, SMS (short message service) text, online chat or through social media (e.g. Facebook). A CIS does not give medical advice and does not replace a doctor, but helps the caller understand prevention, early detection of cancer and their individual situation [6]. The benefits of a CIS are immense especially in developing countries were resource allocation is a constraint. Most successful CIS are dependent on volunteers; information by phone is cheaper than travelling to rural settings, easy to start up and not financially tasking. However, quality-assurance must be maintained to ensure the accuracy of information disseminated [7]. Mobile health (mHealth) is the most important component of CIS and it describes the use of portable electronic devices with software applications to provide health services and manage patient information. With approximately 5 billion mobile phone users globally, opportunities for mobile technologies to play a formal role in health services, particularly in low- and middle-income countries, are increasingly being recognized. Although still limited, there is growing evidence of success in using mobile phones for health (mHealth) to support the performance of health care workers by the dissemination of clinical updates, learning materials, and reminders, particularly in underserved rural locations in low- and middle-income countries where community health workers deliver integrated community case management [8, 9].

Many cancer patients, survivors and their loved ones, often do not know where to go for credible information, especially at the time of diagnosis and this is a major barrier to survivorship, if more patients are informed they would present to the hospital earlier and spend funds on care in the right place [10]. Healthcare professionals are the preferred source of cancer information but their time is limited and a patient’s questions are not usually exhausted in a doctor’s office. Other common sources of information include family and friends, books, the media, and the internet [11]. The quality of information differs depending on the source while some are obsolete and outright detrimental to the public and patient’s health. It is at this point a CIS can offer free, confidential, accurate and up to date information about cancer and support services to cancer patients, their family and friends, the public and health care professionals. In developed countries, CIS are integral components of the national cancer control programme run by the Government and charities [12]. In 1996, theNational Cancer Institute’s (NCI’s) CIS in the United States set up the first CIS and has encouraged thedevelopment of similar CIS programs globally, as evidenced by the formation of the InternationalCancer Information Service Group (ICISG) in 1996 [13]. The ICISG is a worldwide network made up of over 50 organizations that provide free, high quality cancer information services and resources on all aspects of cancer to those concerned or affected by cancer throughout the world. Up till date Sebeccly Cancer Support Centre remains the only Nigerian full member of the ICISG since 2015. The website [14] features a CIS and social media tool box amongst other resources that supports the start-up and or maintenance of a CIS. This study will aim to assess the level of acceptability and utilisation of the mHealth aspects the CIS among the Lagos populace.

## Methods

This is a descriptive study carried out at the Sebeccly Cancer Care (SCC) over a one year period. SCC is a not for profit non-governmental organisation with headquarters in Lagos, Nigeria. The Sebeccly CIS is a health communications program that promotes accurate information to the public, cancer patients and their loved ones. This is done through our online platforms [15–17] and breast help mobilelines (mHealth). This study was limited to the breast help lines only.

An initial period of awareness and sensitisation to cover the entire Lagos metropolitan was carried out in the print and electronic media over a 3 months period by the centre to intimate the public on the scourge of breast cancer and the availability of the breast help lines and means of access. During and after this period, members of the public were given access to our information specialists through the help lineswhich were accessible on weekdays between 10am and 4pm daily except on public holidays. The information specialists who responded to these calls were trained to answer questions, give talks on self-breast examination, counsel, refer and invite callers to the office for breast checks and other related services. They also recorded information of the socio-demographic characteristics and other relevant data of the callers. Descriptive statistics were computed for all relevant data and the data was analysed using Epi info version 7.2 statistical package for windows manufactured by the US Centres for Diseases Control and Prevention. Ethical approval for this study was obtained from the Lagos University Teaching Hospital’s Health Research & Ethics Committee.

## Results

A total of 294 people were reached through the 3 designated help lines manned by the trained cancer information specialists during the nine months study period. Majority [285 (97%)] of the callers were females (Table 1). An age range of 24-55 years was reported with majority being in the 45-49 years age group. Up to 80% of the inquirers were based in Lagos state while 52% were married and 72% had tertiary education. About 90% of the callers owned a mobile phone.

**Table 1:** Socio-demographic characteristics of callers (N=294)

CHARACTERISTICS	FEMALE	MALE	TOTAL
	N_1_ (%)	N_2_ (%)	N (%)
**AGE (YEARS)**			
20-24	8 (2.8)	0 (0.0)	8 (2.7)
25-29	64 (22.5)	1 (11.1)	65 (22.1)
30-34	40 (14.0)	2 (22.2)	42 (14.3)
35-39	5 (1.8)	0 (0.0)	5 (1.7)
40-44	7 (2.5)	1 (11.1)	8 (2.7)
45-49	103 (36.1)	1 (11.1)	104 (35.4)
50-54	32 (11.2)	3 (33.3)	35 (11.9)
55-59	26 (9.1)	1 (11.1)	27 (9.2)
**EDUCATIONAL STATUS**			
Uneducated	4 (1.4)	0 (0.0)	4 (1.4)
Primary	8 (2.8)	0 (0.0)	8 (2.7)
Secondary	35 (12.3)	1 (11.1)	36 (12.2)
Tertiary	156 (54.7)	3 (33.3)	159 (54.1)
Postgraduate	82 (28.8)	5 (55.6)	87 (29.6)
**MARITAL STATUS**			
Single	33 (11.6)	1 (11.1)	34 (11.6)
Married	227 (79.6)	7 (77.8)	234 (79.6)
Widowed	25 (8.8)	1 (11.1)	26 (8.8)
**MOBILE PHONE OWNERSHIP**			
Yes	256 (89.8)	9 (100.0)	265 (90.1)
No	29 (10.2)	0 (0.0)	29 (9.9)
**TOTAL**	285 (100.0)	9 (100.0)	294 (100.0)

As shown in Table 2, majority of the callers (82%) sought information for themselves while the remaining 18% called on behalf of a loved one or friend. For callers who had performed self-breast examinations, majority [248(84.3%)] of callers had no breast abnormality while 38(13%) called to report breast abnormalities and required information on what to do. Only 8 callers (2.7%) were breast cancer patients with pathologic diagnosis who required information on how to live and cope as breast cancer survivors. All the patients with breast abnormalities were either invited to the office for a breast check or referred to a surgery clinic for further evaluation and treatment. Of these 38 women, only 31 women visited the centre and were examined and then referred to the breast surgery clinic. Two (2) were confirmed to be breast cancer while 10 were fibroadenoma; others were lost to follow-up. All the 8 confirmed breast cancer patients were also invited to the center but only 5 patients visited and among who 3 received counselling, navigation services and treatment supports. Of the248 women with no abnormalities, 198 called to understand breast cancer screening guidelines while 100 wanted to be screened (do a mammogram or a clinical breast examination(CBE)) out of which 61 visited the office. A total of 97 callers with no reported breast abnormalities eventually visited the office for breast cancer screening education and services.

**Table 2:** Reasons for calling the help lines (N=294)

REASONS	BREAST ABNORMALITY	NO BREAST ABNORMALITY	TOTAL
	N_1_ (%)	N_2_ (%)	N (%)
To seek personal information	46 (100.0)	195 (78.6)	241 (82.0)
On behalf of a loved one or friend	0 (0.0)	53 (21.4)	53 (18.0)
**TOTAL**	**46 (100.0)**	**248 (100.0)**	**294 (100.0)**

## Discussion

Throughout the world, there is growing interest in people taking more responsibility and involvement in their own health and having information that will enable them to understand how they can maintain a healthy lifestyle [13]. However, there was still a gross underutilisation of the mobile health technology in accessing cancer information as evidenced by the low number of callers recorded in this study despite of the intensive 3 months sensitization period. This may likely be due to the remarkable poverty in the country which has resulted in the lack of interest in the health and health seeking behaviour of the populace.

With the majority of callers being in the 45-49 years age group; this suggests that health seeking behaviour of individuals usually peaks at about the beginning of the middle age although this may not be generalizable within this sample population. It was not surprising that up to 80% of the inquirers were based in Lagos state as the public awareness program was designed to cover only the Lagos metropolitan and thus those inquirers from outside Lagos could possibly have heard of the service from friends or relatives who reside in Lagos state. The high level of education (72% tertiary education) probably confirmed that education plays a pivotal role in the health seeking behaviour of the public. The high number of participants who called the cancer center with their personal mobile phones is also a confirmation of the findings from most studiesthat have suggested the rapid growth of mobile phone users in the low and middle income countries [4, 8–11]. Majority of the callers called primarily to understand cancer prevention and early detection, it is clear that the public seeks information and the breast help lines can bridge the wide gap in cancer education for the public.

In several telephone based CIS studies [5, 18] and the current study; women comprised the overwhelming majority of callers (80%). This can be explained by the fact that women are often the ´gatekeepers´ for health information and healthcare decision-making for their families [19] and also because breast cancer is predominantly a female disease which may thus cause more enquiries to emanate from the female gender.

Since mHealth seems to be employed only for limited uses and during limited phases of the care process [4, 8, 9] as it had been demonstrated in this study where only a few callers were recorded to have accessed the system during the study period. Therefore, it is unlikely that it can really contribute to the creation of new care models in the short-term. This under-utilization may depend on many issues, including the need for it to be embedded into broader information systems through policy formulation by creatinga higher level of targeting at all stages and in all health care delivery activities and not just in cancer care. A major limitation of the study was the inability to ascertain the actual number of intending callers who the center may have missed their calls especially during the non-working days of the week or those who are willing to do so but not able to have access to a telephone line.

## Conclusion

There is still a gross lack of awareness and underutilisation of the CIS in Nigeria. Therefore the rapid growth of mobile phone use in the Nigeria has presented a unique opportunity and promise where the formation of partnerships between governments, technologists, non-governmental organizations, academia, and industry, can provide a great potential to improve cancer care via the use of mHealth. By harnessing the increasing use of mobile phones among diverse populations, there is promising evidence to suggest that mHealth can be used to deliver increased and enhanced health care services to the increasing population of cancer patients in the country. However, as with many other health improvement projects, a key challenge will be on how to move the mHealth approach from a regional pilot project to a national scalable program while properly engaging health workers and communities in the process. It is therefore imperative that a more robust national pilot program should be carried out to assess the acceptability and utilisation of this laudable innovation.

### What is known about this topic

- A Cancer Information Service (CIS) is a system created to provide the cancer information needs such as patient education, emotional support and counselling services to people affected by cancer;
- A CIS can be provided by phone (mobile health), email, SMS (short message service) text, online chat or through social media (e.g. Facebook);
- Mobile health (mHealth) is the most important component of CIS and it describes the use of portable electronic devices with software applications to provide health services and manage patient information.

### What this study adds

- That there is still a gross lack of awareness and underutilisation of the CIS in Nigeria compared to the developed world;
- That instituting regular public awareness programs may increase the level of awareness and utilisation of the CIS;
- That the increasing growth of mobile phones users among the populations has presented a unique opportunity to deliver enhanced health care services to the increasing population of cancer patients in the country.
